# Is 50 cent the price of the optimal copayment? - a qualitative study of patient opinions and attitudes in response to a 50 cent charge on prescription drugs in a publicly funded health system in Ireland

**DOI:** 10.1186/1472-6963-13-16

**Published:** 2013-01-10

**Authors:** Sarah-Jo Sinnott, Marie Guinane, Helen Whelton, Stephen Byrne

**Affiliations:** 1 Department of Epidemiology and Public Health, University College Cork, Cork, Ireland; 2 Pharmaceutical Care Research Group, School of Pharmacy, University College Cork, Cork, Ireland; 3 Oral Health Services Research Centre, University Dental School, Wilton, Cork, Ireland

**Keywords:** Qualitative research, Copayment, Health policy, Adherence, Ireland

## Abstract

**Background:**

A 50 cent prescription levy was introduced in 2010 on the General Medical Services (GMS) scheme (Irish public health insurance). This study sought to examine patient attitudes and opinions surrounding the 50 cent copayment. Given the small momentary value of the prescription fee, these results are of interest to policymakers internationally who wish to reduce copayments rather than abolish them.

**Methods:**

A qualitative research design was used; semi structured interviews were carried out. Twenty four GMS eligible participants were interviewed in 23 interviews. Fifteen females and 9 males took part. Ages varied from 31- >70 years. Patients were invited to be interviewed in both independent and chain community pharmacies in three types of setting; 1) a socially deprived urban area, 2) a suburban affluent area and 3) a rural area. The Framework method was used for data management and analysis using QSR International’s NVivo 9.2 qualitative data analysis software. The “Francis method” was used to test for data saturation.

**Results:**

Results are of interest to the Irish context and also at a broader international level. Patients were mostly accepting of the prescription levy with some reservations concerning an increased price and the way in which generated revenue would be used by government. Participants identified waste of prescription drugs at the hand of patients (moral hazard), but there was discordant opinion on whether the 50 cent copayment would halt this moral hazard. Interviewees felt the levy was affordable, albeit some may suffer a financial impact more than others.

**Conclusions:**

This qualitative study gives important insights into the experiences of GMS patients with regard to the prescription levy. Information regarding the appropriateness of a 50 cent copayment as a symbolic copayment needs to be confirmed by quantitative analysis. Further insight is required from a younger population.

## Background

The General Medical Services (GMS) scheme in Ireland is a tax funded, means tested, public health insurance scheme [1]. The GMS scheme provides free hospital care, GP services and prescription drugs to those who qualify. Means tested thresholds differ for those under and over 70 years, which results in almost 100% of those over 70 years qualifying, along with low income and social welfare dependent individuals [2]. Currently, 38% (1,615,809) of the Irish population receive healthcare on this scheme [3]. The remainder of the Irish population are deemed to be “private” patients who either have private health insurance plans or who pay out of pocket for healthcare. Private health insurance plans in Ireland do not entitle the holder to free GP care or prescriptions. Instead, “private” patients can enroll on the government provided Drugs Payment Scheme (DPS) which allows patients to purchase drugs up to the value of €132/month per family. After this ceiling is passed, the remainder is reimbursed.

Due to rising expenditure in the GMS drug budget, a copayment of 50 cent per prescription item was introduced in October 2010, which was capped at €10 per household monthly. Copayments generate cost savings via reductions in moral hazard, which is wasteful use of a good at the expense of a third party payer. In addition to savings, additional revenue can be generated [4]. However, copayments are not without their disadvantages. Copayments are associated with decreased utilisation of medicines; this decrease varies in magnitude according to cost of copayment and also whether medicines are regarded as essential or less-essential, the use of the latter being affected more [5-8]. Essential medicines are generally understood to be treatments that are important in disease progression and in life prolongation, whereas less-essential drugs play a role solely in symptom control [9]. Therefore it can be appreciated how copayments are implicated in public health concerns; the reduced use of essential medicines has been associated with negative health outcomes for patients [9-12]. Poor clinical outcomes result in increased use of health services and increased expenditure overall [9-13].

Recently, there has been a move towards reducing financial barriers for essential medicines. This has been done via creation of value-based insurance design in America [14] and removal of copayments for all medicines in Scotland, Wales and Northern Ireland [15,16]. However, full coverage for prescription medicines is linked with induced moral hazard [17]. Therefore small, “symbolic” copayments have been advocated [18]. Such copayments would, in theory, represent a disincentive to partake of medicines that are not necessary, while remaining cheap enough to facilitate continued adherence to essential medicines.

Two qualitative studies of copayments for medicines on the National Health Service (NHS) in the UK have been carried out [19,20]. Both papers give valuable insights into general opinions of cost-sharing and also how patients deal with cost of medicines. The NHS and GMS are similar in that both are tax funded public health insurances. The populations of the UK and Ireland are also culturally similar. However large differences exist; first the copayment is much bigger on the NHS (£7.65 in 2012 in England). Secondly, the NHS provides healthcare to the entire population whereas patients who qualify for the GMS in Ireland would generally be exempt from NHS prescription charges; therefore their views have not been captured to date. An Italian copayment of €1.70 has been explored in a cross country comparison between the NHS of the UK and Italy [21]. However, as with the UK NHS, the Italian NHS provides whole population coverage, in contrast to the two-tiered health system in Ireland. Despite this, it is interesting to note that Italians in this study reported less affordability problems than UK patients. An Australian qualitative study explored moral hazard, however again the copayments are dissimilar to the Irish copayment with pensioners paying $3.80 for prescriptions in 2004 [22]. Furthermore, an increase in this copayment in 2005 led to decreased dispensings of some essential drugs; an unwanted feature of a limited copayment policy [23]. Some American qualitative studies have been carried out, however, they were focused on the logistical challenges that patients were faced with when Medicare Part D was established,[24] concentrated on how patients managed their medicines when taking multiple drug regimens [25] or were concerned solely with the opinions of mental health patients [26]. Even though there is an extensive literature available on the quantitative effects of copayments, and how this varies according to price paid and medicines used, there is a limited qualitative literature on examining public attitudes to small copayments. The “symbolic” nature of the Irish copayment may be particularly true given its small value in comparison to copayments in other European countries [27,28].

The aim of this study is to address this gap in the literature by exploring the opinions of GMS patients around whether 50 cent could be an appropriate limited or “symbolic” copayment. Additionally, general opinion and attitudes are investigated to gain relevant information for Irish policy development.

## Methods

### Ethics

Ethical approval was sought from and granted by the Clinical Research Committee of the Cork Teaching Hospitals prior to initiating the study.

### Interviewing method

This study is part of a larger project that will also qualitatively examine the views and opinions of GPs and pharmacists and encompass a quantitative element using a large national prescribing dataset. Therefore a qualitative approach was taken in this instance to complement the quantitative data; giving meaning and explanation for any quantitative patterns observed.

Interviewing was chosen as the data collection method for several reasons. First, issues around money and medicines can be perceived as delicate topics; therefore discussion in a focus group may be stilted. Secondly, interviews provide advantages over focus groups in that in depth coverage of a subject can be achieved as opposed to wider superficial coverage in a focus group [29].

Interviews took place between January and March, 2012.

### Participants

The sampling frame for this study was all GMS patients attending a community pharmacy in the province of Munster, Ireland. Patient views from 3 types of location were required; an affluent urban area, a socially disadvantaged urban area and a rural area [30]. This sampling frame was intended to capture opinions across a range of social circumstances, ages and gender. It was necessary to locate pharmacies that were independently owned and those that were part of a chain group, as there could be subtle demographic differences in attenders at both types of pharmacy (Table 1). Through personal contacts 5 pharmacists were invited to participate in the study, all agreed to be involved. Once pharmacist participation was agreed, an interviewing date was agreed upon for one of two interviewers to attend the pharmacy. Patients were conveniently sampled from purposively sampled pharmacies [29]; that is pharmacists invited patients who presented at the pharmacy on a given day to participate. Selection was not based on prior knowledge of patient opinion towards the charge or as representation of the pharmacist viewpoint. Patients were free to decline participation in the study. One patient did refuse inclusion in study because she did not wish to be interviewed. This demonstrates patient autonomy in participation and also minimization of selection bias. Once patients agreed to be interviewed, the interviewer explained who they were, clarified the aims and objectives of the study and assured participants of anonymity and data confidentiality. Participants were asked for verbal consent which was recorded using a Dictaphone.

**Table 1:** Distribution of Pharmacies by location and pharmacy ownership status

	**Independent pharmacy**	**Chain pharmacy**	**Total**
Affluent Urban		1	1
Socially Deprived Urban	1	1	2
Rural	1	1	2
Total	2	3	5

### Data collection

A topic guide for semi structured interviews was developed to gain information relevant to the study objective, drawing on the existing literature and professional experience. Themes that were identified to be discussed included knowledge of levy, opinion of levy, rational use of medicines in relation to the levy and suggestions for future policy formation. The guide was piloted with 2 pharmacists who advised modifications based on their experiences with patients. Table 2 summarises the final themes discussed. Interviews took place in pharmacies instead of patient homes for reasons of access and expedience. Pharmacy consultation areas provided confidential spaces to conduct interviews. Some interviews were conducted in pharmacy seating areas, however it is not anticipated that this had a negative impact on the quality of data obtained. Interviews ranged in time from roughly 5 minutes to more than 30 minutes.

**Table 2:** Themes discussed in interviews


Demographic information	
Knowledge of the purpose of the levy	What do you know about the 50 cent levy?
Opinion on levy	What did you feel when the government introduced a 50 cent levy on your prescription drugs?
Waste of prescription drugs	What is your impression of waste when it comes to prescription drugs?
Effect of levy on medicine utilization	Does the levy make you think any differently about collecting your medicines?
The levy as a barrier to utilization	
Prioritisation of medicines	Why would you leave some medicines out?
Effect of not taking medicines on health	Are you aware of any side effects of not taking medicines?
Suggestions for policy	

No rigid rules exist for determining sample size in a qualitative study. Generally, interviews are carried out until no new material occurs in interviews, defined as data saturation. While this is an accepted method in qualitative research, there is no definitive test for saturation of data. Francis *et al.* have proposed a method for detecting data saturation [31]. This method involves, first, identifying an “initial analysis sample” based on appropriately sized sample sizes and second, defining the “stopping criterion” – e.g., how many additional interviews will be carried out with no new themes emerging, at which point data saturation can be declared. It was decided that 20 interviews would be undertaken in this study and then an additional 3 interviews were carried out to test for data saturation. Data saturation appeared to have occurred by the 19^th^ interview, however interestingly new material arose in the 20^th^ and 23^rd^ interviews. Therefore, the traditional method of determining sample size would have seen recruitment stop at the 19^th^ interview; however the Francis method in this case suggested further recruiting until 3 consecutive interviews with data saturation were obtained. Due to reasons of practicality, further interviews were not conducted. It was taken that repetition of themes in the 21^st^ and 22^nd^ interviews was adequate to satisfy traditional requirements of data saturation.

### Analysis

The Framework approach was used to manage and analyse data, because its use is applicable in policy research [29]. Data (transcripts) were inputted into QSR International’s NVivo 9.2 qualitative data analysis software [32]. The Framework method consists of five steps. The first step of data familiarisation (reading and re-reading) occurred from a very early stage due to data transcription. The second step was drawing up a preliminary thematic framework, which was informed by the topic guide and emergent themes from interviews. This framework was revisited and amended appropriately when all interviews were completed. The framework was then applied to the data and “tested for fit” which is the third step in the framework method. The fourth stage involved the data being charted (sorted and synthesised) on large matrices. Language used by participants was used as much as possible at this stage to retain meaning and context in the data. The last stage involved a more abstract process of assigning higher meaning to charted data, pulling together core themes and providing associations and explanations for these. Memo writing, while advocated as a large part of Grounded Theory, was used in this analysis [33]. The function of memo writing was to facilitate reflections on the data and provide an audit trail of decision processes.

### Intra-coder reliability

A sample of 3 random indexed manuscripts was verified by MG for accuracy of the thematic framework and application of the framework to the transcripts. Some disagreements in coding arose. The most common reason for disagreement was redundant themes for the same phenomenon e.g., empathy and relative affordabilities. Through discussion these indexing discrepancies were remedied [34].

## Results

Twenty four people were interviewed (Table 3). One of the 23 interviews involved 2 people, a husband and wife. The dialogue between them added to the quality of the data. Three different types of location were sampled from in order to gain a spectrum of opinions.

**Table 3:** Characteristics of interviewees

**Sex**	**Male**	**9**
	**Female**	**15**
Frequency of age groups	31-40	1
	41–50	2
	51–60	8
	61–70	3
	>70	10
Geographic location	Urban Deprived	8
	Urban Affluent	4
	Rural	12
Level of education	Primary School	13
	Secondary School	7
	Third Level	2
	Special School	1
	Missing Data	1
Private health insurance	Yes	4
	No	19
	Missing Data	1
Employment status	Sheltered employment	1
	Part Time Employment	2
	Retired	10
	Housewife	1
	Unemployed	10
Length of time eligible for medical card	<2 yrs	1
	2-5 yrs	5
	6-10 yrs	4
	11-15 yrs	4
	15-20 yrs	2
	25-30 yrs	2
	>30 yrs	3
	Missing data	3
Number of medicines	1-2	5
	3–4	3
	5–6	5
	7–8	4
	9–10	4
	11–12	1
	13–14	1
	>15	1
Types of medicines	Oral hypoglcaemics, anti-hyperlipidaemics, anti-hypertensives, aspirin, nitrates, psychotropics, painkillers, immunomodulators, osteoporotic agents, bronchodilators and inhaled steroids, gastric acid suppressants.	

The results of this study are spilt into 2 domains. The first domain refers to how participant opinions and insights relate to the Irish context. The second domain alludes to how symbolic copayments could play a role in reducing moral hazard, as perceived by members of the GMS population, without implicating patient adherence to important medicines.

### Context specific to Ireland

#### General views on prescription charges

Generally, interviewees were supportive of the levy. There was an overarching conditional acceptability; patients appeared to be accepting of the charge, but would like raised revenue to be used constructively. “*Well it’s a good idea if there’s something good going to be done with it like. As long as it’s not just swallowed up with everything else” ***04FJ.**

In this group there was an underlying element of appreciation for their medical cards “*we’re lucky we have our medical card” ***06FJ.** There was an awareness of financial pressure within the government and this appeared to ameliorate any potential feelings of unjustness with the policy. Those who disagreed with the 50 cent levy were strongly opposed to it, not for reasons of affordability, but for reasons of principle such as inequity and a sense of entitlement *“After working all my life as a xx…. I think I was after working for the medical card”***14MC.** Issues of affordability arose when the levy was discussed in combination with other recent charges and levies in Ireland. Interestingly, some of those who were opposed to the levy were located in rural and affluent areas, as opposed to socially disadvantaged areas.

### Small copayments as symbolic contribution

#### Patient determined causes of prescription medicine waste

A large proportion of patients identified the presence of moral hazard within the realm of prescription drug supply and demand. Many patients made references to people who collect tablets they do not use properly or do not need, allowing them to pile up at home. There was a feeling that the prescription levy could play a role in reducing this type of waste “*I think with the levy now, people are getting more clued in, when they have to pay. I think it’s a great thing. Because people can say “oh God, I don’t need that – I have that at home”***10FC.** Some participants were cognoscente of the fact that the levy intentionally serves to diminish moral hazard and perceived this positively *“…Cos people would get them for nothing, they‘d just throw them there and take what they want. They’ve no responsibility like for what ‘tis costing. So I think it’s a good thing in there…” ***07 MJ.**

There was conflicting opinion on whether the 50 cent levy was effective in reducing this type of moral hazard. It was acknowledged that some people who waste medicines will continue to attend their pharmacy monthly because of habit, forgetfulness or perhaps because of loneliness. The opinion in this instance was that the 50 cent levy was not of sufficient financial worth to halt wasteful use of medicines *“if you’re the type that’s going to stock up on pills the fifty cents isn’t going to stop you” ***05FJ.** In addition to this, the notion of a financial cost adding value and encouraging use of medicines that were previously wasted was disagreed with “*but I mean, if you need medication, this 50 cent per prescription doesn’t really make the difference between whether that forces ya or encourages you to take it or not. Not 50 cent, no” ***03 MJ**

#### Other causes of prescription medicine waste

Conscientious prescribing was mentioned as a determining factor in accessibility to medicines. Most people trusted that they would not be prescribed medications unless they needed them and trusted their doctors to make rational choices for them*“….Like I was told I’d get now, since I got sick – if I was told to stand on my head three times a day I would do it…” ***05FJ**

Potential waste in prescribing was described. Some people mentioned that automatically generated prescriptions often had unnecessary items on them. There was a feeling that prescriptions should be subject to doctor review and that the need for items on a prescription should be discussed with the patient – in this way identification of drugs that are not working, or drugs that patients have stopped using could occur, thus reducing waste.

"“….should be a review anyways….in terms of what the doctor has on his records, and say ok “let’s do a clean up here”, emmm “do you need this?”, “you don’t really”. But…… sometimes and I know for a fact….that there isn’t a clean-up and it’s just happening willy-nilly. So maybe if there was better practise in terms of the doctor-patient interchange………then I think it would tidy up a lot of excess……” **24MD.**"

There was a sense that the 50 cent levy should in theory encourage this type of prescription review by GPs, but that in reality it does not.

#### Theorised negative effects of levy

In general, the 50cent levy did not appear to affect patient adherence to medicines, despite some being vehemently opposed to the policy. One patient did stop taking her cholesterol tablets for a period due to the cost of the levy, however has since started to take them again – this was thought to be a response to the principle of the levy as opposed to the cost of it. Conversely, others reported that they would sacrifice other goods first before not taking their medicines *“…I wouldn’t be able to go without my medication. I’d just have to actually cut back on my food, or cut back on my household…” ***06FJ.**

#### Affordability of symbolic copayments

Overall there appeared to be limited problems with affordability of the 50 cent copayment. Most participants found the 50 cent to be financially acceptable – some trivialised the cost comparing it to less than the price of a bar of chocolate. There were 2 individuals, who although did not explicitly admit financial difficulty with the 50 cent levy, did discuss financial hardship repeatedly. “*Well considering I’m on a low budget in a council house…. I think t’was a bit harsh you know what I mean, they should look at peoples’ backgrounds as well as their medical conditions and just say, you know, help them up” ***17FC.**

While financial hardship was mentioned in many of the interviews; affordability issues seemed to revolve around rising costs and charges for household bills, potential septic tank inspections, and cutbacks in allowances for household heating. Therefore, disgruntlements may be more associated with austerity at a broader level as opposed to the 50 cent levy in particular.

"“Well now it’s not the levy itself, it’s the other things that are a tenner here, for my pension now, and a tenner there, and a tenner everywhere. It’s not the medicine, I’ve no qualms about paying for medicine”**07 MJ**"

#### Recommendations

The majority of interviewees felt that the 50 cent was a reasonable amount for the levy, and that if it was left at this people would continue to afford it. Those who were opposed to the levy felt that it should be abolished. An interesting theme of increased use of doctor services arose on discussion of abolishing the levy.

*"“……I’d probably be going to the doctor more often with smaller things, you know. This way you don’t like, you try to avoid the doctors like….” ***16FC**

When asked about the possibility of a raised levy, most people felt that a euro per item would be acceptable whereas anything ranging from €2-€5 would be prohibitive. There was an emergent sense of empathy as people referred to how a raised levy would be unfair on those with chronic illness. A very small number of people said that their adherence to their medicines would be affected by a raised charge, but most felt that their medicines were too important to stop taking.

*"“……I would have to take my medication because top doctors have warned me like. So I would have to take my medication on a regular basis……” ***19MC**

## Discussion

This exploratory study provides a range of insights into patients’ views on the 50 cent prescription levy. General opinion and attitudes, cost-coping behaviours and awareness of moral hazard, which are relevant to the Irish context, echo those already found in the literature, even those studies examining more expensive copayments [19-22]. The finding from this qualitative research of most interest to Irish policymakers is that the majority of patients appear to have accepted the prescription levy. Patients seem to accept the levy because they are aware of increased financial pressure within the health services and acknowledge that wasteful use of drugs contributes to this problem, a feature that has also been observed in international populations [20,35]. A sense of entitlement to free medicines, after payment of taxes, was expressed, again this has been reported elsewhere [20]. However, the American literature highlights the fact that small copayments do not impinge on perception that patients’ medical needs are being catered for, a theme repeated in this study nut only in those who are supportive of levy [35]. Interviewees spoke at length about financial hardship and expressed concern about rising charges and increasing prices of household bills. However, worries were related to the accumulation of charges as opposed to the prescription levy in isolation.

Regarding the appropriateness of 50 cent as a suitable symbolic copayment, it is worthwhile examining if patients have experienced the negative implications of cost-sharing, such as reduced adherence. In this qualitative study of 24 participants, patients perceive necessity of treatment to be more important than the cost of the medicines to them. In addition, patients forecast the negative effects of not adhering to medications and this also appears to be a bigger determinant of medication adherence than the 50 cent levy. That patients do not allow cost alone to influence their medicine taking behaviour echoes previous qualitative findings [19,22,25]*.* However, given that this is a qualitative study – these results cannot be generalised to whole populations especially in light of a plethora of large scale quantitative studies which point to copayments effecting a reduction in utilization [5,7]. Furthermore, quantitative evidence points to reductions in utilisation seen in vulnerable populations i.e., the poor, the elderly and the chronically ill; groups which comprise the GMS population in Ireland [36-39].

A review article by Eaddy *et al.,* shows the negative relationship between price paid for medicines and adherence [8]. While copayment policies aim to reduce the use of unnecessary medicines, there is a contemporaneous reduction in the use of essential medicines resulting in worsening patient adherence, subsequent poor health outcomes and increased consumption of the health services [9-13]. Therefore, an ideal copayment would achieve an optimal balance between a pecuniary value to halt moral hazard associated with less-essential medicines and a value which does not act as a disincentive to adherence to essential medicines. This type of copayment would be advantageous in that it can reduce government expenditure on unnecessary drug use, while generating “bonus” income from copayment charges. The Beveridge type model of care that is expected from patients [20,35] can also be maintained with such copayments. Thus, from an international perspective, these results are of interest considering the small value of the copayment and how this may relate to recent advocacy for limited copayments [18,40].

The results of this study reveal inconclusive evidence for 50 cent being an approriate price of a copayment. Participants gave conflicting opinions on whether 50 cent would halt moral hazard. Copayments work on the premise that patients make rational decisions about what medicines are less-essential. However, patients in this study were reluctant to prioritise medicines and relied on their doctors to make rational decisions for them. Patients appeared to accept that if a doctor prescribed something then it was necessary to take, this patient view has also been expressed elsewhere [19,22]. Therefore, there may be a role for health professionals such as general practitioners and pharmacists to encourage patients to review their medicines; indeed 1 patient proposed medicines use reviews (MURs). MURs seek to increase patients’ understanding of their medicines, reduce medication related morbidity, and improve appropriate medicine taking. However, the evidence for pharmacist led MURS in contracting costs is not encouraging and they are sometimes met with patient resistance [41,42]. Despite the poor evidence for pharmacists led MURS, the issue of prescribers being subject to moral hazard has been discussed in the literature [20,43] and is a factor that should be considered both in policy development and by individual prescribers.

Most patients said they would not give up their medicines due to a hypothetical price increase. It has been suggested that patients who think they would forgo a tablet because of clinical reasons e.g., unpleasant side effects, actually in real world circumstances stop taking their tablets due to practical reasons such as cost [25]. Therefore patient recommendations to increase the prescription levy should be taken with heed. Likewise, recommendations to abolish the levy could induce increased use of doctor services, along with a loss of the associated financial advantages.

### Limitations

Patients of a young age were not recruited to the study. This may have occurred because data collection occurred during work hours. This limitation demonstrates a gap in the data collected, and highlights the need to include younger individuals in future qualitative studies on this topic.

As mentioned in the methods section, a convenience sample was used for data-collection. Concerns regarding selection bias in recruitment are mediated by the fact that the sample obtained is representative of the GMS population, with a high number of older individuals involved [44]. Patients were not fore-warned about interviews taking place and so did not have opportunity to think about the topic beforehand. This may have limited the quality of the data and resulted in short interviews. However, 23 interviews were carried out which limits the impact of this drawback. In addition, interviews were carried out in community pharmacies which are not a conventional venue choice. Data acquisition was thought not to be affected by venue type as opinions across a broad spectrum were gathered; as opposed to one dominant opinion which would be expected if individuals felt prohibited from expressing themselves truthfully in this situation. Furthermore, holding convenience interviews in a pharmacy setting removed logistical barriers for participants.

Individuals who choose not to collect their prescriptions because of the prescription charge cannot be sampled and interviewed in a pharmacy setting. However, an interview did take place with one individual who had stopped taking her medicine in the past due to the cost; therefore insight into this situation was gained. Other studies may choose to sample from a GP waiting room to capture those that attend their GP but are primary non-adherers [45]. However, as one interviewee pointed out; when the prescription charge is in place people may choose to avoid attending their doctor also.

The main researcher is a pharmacist and the second interviewer was a final year pharmacy student, therefore there was a possibility that patients gave socially desirable responses. This bias was difficult to eliminate as the research team felt that by disclosing the researchers’ backgrounds to interviewees an element of professionalism would be introduced into the interviews. It is difficult to test for the presence of this bias. However given that one participant felt comfortable in discussing her non-adherence to cholesterol lowering medicine, it may be taken that the interviewers established a solid rapport with participants and socially desirable answers did not feature dominantly in this study.

## Conclusions

The 2001 health policy document Quality and Fairness – A health system for you [46] outlined that the Irish health system would be one that “*encourages you to have your say, listens to you, and ensures that your views are taken into account”*

This study has allowed patients to express their views and experiences with the 50 cent prescription levy. For the most part, patients are willing to accept the copayment. Patients have made valuable suggestions along with highlighting areas that should be addressed to achieve higher efficiency in controlling the drug budget, such as rational prescribing. According to promises laid out in Quality and Fairness, these views should now be incorporated into any further policy development. Further interviews with younger GMS patients and primary non-adherers are required to capture the picture of the broader population.

On a broader scale, the results of this study give some direction in the search for the optimal copayment. There was discordant opinion on whether 50 cent can halt moral hazard. However, this should be considered along with the fact that participants seem reluctant to sacrifice medicines at this lower end of copayment scale. There may be flexibility for increasing the levy. However, such a policy move should first be supported by a large scale quantitative analysis to assess the differential effect of the 50 cent copayment on essential and less-essential drugs before drawing any final conclusions on its appropriateness.

## Competing interests

The authors declare that they have no competing interests.

## Authors’ contributions

SJS, HW and SB conceived of the study. SJS, MG and SB decided on sampling techniques. SJS and MG carried out data collection. SJS analysed and interpreted data. MG carried out verification of data analysis. SJS wrote the final manuscript; MG, HW and SB revised manuscript. All authors read and approved the final manuscript.

## Pre-publication history

The pre-publication history for this paper can be accessed here:

http://www.biomedcentral.com/1472-6963/13/16/prepub

## References

[B1] McDaid D Wiley M Maresso A Mossialos E Ireland: Health system review. Health Systems in Transitionvol. 11. Copenhagen Ø 2009 Denmark: World Health Organization 2009, on behalf of the European Observatory on Health Systems and Policies 1 268

[B2] Central Statistics Office Women and Men in Ireland 2011 Dublin, Ireland: Stationery Office

[B3] Health Service Executive Primary Care Reimbursement Service Statistical Analysis of Claims and Payments 2010 2010 pg4. Available from: http://www.hse.ie/eng/staff/PCRS/PCRS_Publications/claimsandpayments2010.pdf.

[B4] McPake B Normand C Health economics: an international perspective -2nd ed 2008 Routledge

[B5] Austvoll-Dahlgren A Aaserud M Vist GE Ramsay C Oxman AD Sturm H Kösters JP Vernby A Pharmaceutical policies: effects of cap and co-payment on rational drug use 2008 Cochrane Database of Systematic Reviews 2008, Issue 1. Art. No. CD007017 10.1002/14651858.CD007017 18254125

[B6] Gemmill MC Thomson S Mossialos E What impact do prescription drug charges have on efficiency and equity? Evidence from high-income countries Int J Equity Health 2008 7 12 10.1186/1475-9276-7-12 18454849 PMC2412871

[B7] Goldman DP Joyce GF Zheng Y Prescription drug cost sharing associations with medication and medical utilization and spending and health JAMA 2007 298 1 61 69 10.1001/jama.298.1.61 17609491 PMC6375697

[B8] Eaddy MT Cook CL O’Day K Burch SP Cantrell CR How patient cost-sharing trends affect adherence and outcomes: a literature review P T 2012 37 1 45 55 22346336 PMC3278192

[B9] Tamblyn R Laprise R Hanley JA Abrahamowicz M Scott S Mayo N Hurley J Grad R Latimer E Perreault R Adverse events associated with prescription drug cost-sharing among poor and elderly persons JAMA 2001 285 4 421 429 10.1001/jama.285.4.421 11242426

[B10] Hsu J Price M Unintended consequences of caps on Medicare drug benefits N Engl J Med 2006 354 22 2349 2359 10.1056/NEJMsa054436 16738271

[B11] Soumerai SB Ross-Degnan D Avorn J McLaughlin T Choodnovskiy I Effects of Medicaid drug-payment limits on admission to hospitals and nursing homes N Engl J Med 1991 325 15 1072 1077 10.1056/NEJM199110103251505 1891009

[B12] Soumerai S Thomas JM Ross-Degnan D Casteris CS Bollini P Effects of limiting Medicaid drug-reimbursement benefits on the use of pychotropic agents and acute mental health services by patients with schizophrenia N Engl J Med 1994 331 10 650 655 10.1056/NEJM199409083311006 8052275

[B13] Sokol MC McGuigan KA Verbrugge RR Epstein RS Impact of medication adherence on hospitalization risk and healthcare cost Med Care 2005 43 6 521 530 10.1097/01.mlr.0000163641.86870.af 15908846

[B14] Choudhry NK Fischer MA Avorn J Schneeweiss S Solomon DH Berman C Jan S Liu J Lii J Brookhart MA At Pitney Bowes, value-based insurance design cut copayments and increased drug adherence Health Aff (Millwood) 2010 29 11 1995 2001 10.1377/hlthaff.2010.0336 21041738

[B15] Groves S Cohen D Alam MF Dunstan FD Routledge PA Hughes DA Myles S Abolition of prescription charges in Wales: the impact on medicines use in those who used to pay Int J Pharm Pract 2010 18 6 332 340 10.1111/j.2042-7174.2010.00063.x 21054593

[B16] National Health Service Free Prescriptions and Charges for Drugs and Appliances) (Scotland) Regulations 2011. Scotish Parliament, Scottish Statutory Instruments,55 2011 Available from; http://www.legislation.gov.uk/ssi/2011/55/pdfs/ssi_20110055_en.pdf.

[B17] Eisenhauer JG Severity of Illness and the Welfare Effects of Moral Hazard Int J Health Care Finance Econ 2006 6 4 290 299 17136599 10.1007/s10754-006-9006-3

[B18] Asato J Charging Ahead? 2006 The Social Market Foundation: Spreading the Costs of Modern Public Services. In

[B19] Schafheutle EI Hassell K Noyce PR Weiss MC Access to medicines: cost as an influence on the views and behaviour of patients Health Soc Care Community 2002 10 3 187 195 10.1046/j.1365-2524.2002.00356.x 12121255

[B20] Schafheutle EI Patients’ views on the UK policy of prescription charges–Insights from qualitative interviews Res Social Adm Pharm 2008 4 4 343 354 10.1016/j.sapharm.2008.02.001 19064241

[B21] Atella V Schafheutle E Noyce P Hassell K Affordability of medicines and patients’ cost-reducing behaviour: Empirical evidence based on SUR estimates from italy and the UK Appl Health Econ Health Policy 2005 4 1 23 35 10.2165/00148365-200504010-00005 16076236

[B22] Doran E Robertson J Henry D Moral hazard and prescription medicine use in Australia–the patient perspective Soc Sci Med 2005 60 7 1437 1443 10.1016/j.socscimed.2004.08.005 15652677

[B23] Hynd A Roughead EE Preen DB Glover J Bulsara M Semmens J The impact of co-payment increases on dispensings of government-subsidised medicines in Australia Pharmacoepidemiol Drug Saf 2008 17 11 1091 1099 10.1002/pds.1670 18942671

[B24] Perry M Kitchman M Guyer M The Medicare Rx Drug Law. Medicare’s new prescription drug benefit: the voices of people dually covered by Medicare and Medicaid 2005 Washington, DC: The Henry J. Kaiser Family Foundation 12. Publication 7243. Available from: http://www.kff.org/medicare/upload/Medicare-s-New-Prescription-Drug-Benefit-The-Voices-of-People-Dually-Covered-by-Medicare-and-Medicaid-Report.pdf.

[B25] Elliott RA Ross-Degnan D Adams AS Safran DG Soumerai SB Strategies for coping in a complex world: adherence behavior among older adults with chronic illness J Gen Intern Med 2007 22 6 805 810 10.1007/s11606-007-0193-5 17406952 PMC2219857

[B26] Hensley MA Policy Implications of Medicare Part D for Adults With Mental Illness: A Qualitative Exploration Soc Work Public Health 2012 27 3 238 249 10.1080/19371918.2011.542975 22486429

[B27] Noyce PR Huttin C Atella V Brenner G Haaijer-Ruskamp FM Hedvall M Mechtler R The cost of prescription medicines to patients Health Policy 2000 52 2 129 145 10.1016/S0168-8510(00)00066-X 10794841

[B28] The Scottish Executive Review of prescription charges in western Europe, North America and Ausralasia 2006

[B29] Ritchie J Lewis J Qualitative Research Practice A Guide for Social Science Students and Researchers – Chapter 9 “Carrying out Qualitative Analysis 2008 Sage Publications: London

[B30] Haase T Pratschke J Pobal HP Deprivation Index - (The 2011 Pobal HP Deprivation Index for Small Areas (SA) Available from: http://trutzhaase.eu/deprivation-index/the-2011-pobal-hp-deprivation-index-for-small-areas.

[B31] Francis JJ Johnston M Robertson C Glidewell L Entwistle V Eccles MP Grimshaw JM What is an adequate sample size? Operationalising data saturation for theory-based interview studies Psychol Health 2010 25 10 1229 1245 10.1080/08870440903194015 20204937

[B32] NVivo qualitative data analysis software QSR International Pty Ltd. Version 9.2

[B33] Corbin JM Strauss A Grounded theory research: Procedures, canons, and evaluative criteria Qual Sociol 1990 13 1 3 21 10.1007/BF00988593

[B34] Carey JW Morgan M Oxtoby MJ Intercoder agreement in analysis of responses to open-ended interview questions: Examples from tuberculosis research Field Methods 1996 8 3 1 5 10.1177/1525822X960080030101

[B35] Bernstein J Stevens RA Public opinion, knowledge, and Medicare reform Health Aff (Millwood) 1999 18 1 180 193 10.1377/hlthaff.18.1.180 9926655

[B36] Rice T Lavarreda SA Ponce NA Brown ER The impact of private and public health insurance on medication use for adults with chronic diseases Med Care Res Rev 2005 62 2 231 249 10.1177/1077558704273806 15750178

[B37] Rice T Matsuoka KY The impact of cost-sharing on appropriate utilization and health status: a review of the literature on seniors Med Care Res Rev 2004 61 4 415 452 10.1177/1077558704269498 15536208

[B38] Lexchin J Grootendorst P Effects of prescription drug user fees on drug and health services use and on health status in vulnerable populations: A systematic review of the evidence Int J Health Serv 2004 34 1 101 122 10.2190/4M3E-L0YF-W1TD-EKG0 15088676

[B39] Gemmill MC Thomson S Mossialos E What impact do prescription drug charges have on efficiency and equity? Evidence from high-income countries Int J Equity Health 2008 7 12 10.1186/1475-9276-7-12 18454849 PMC2412871

[B40] Schafheutle E Comment-Agenda for 2006-//Is limiting rather than abolishing prescription charges the answer? Pharm J 2006 276 7394 388 389

[B41] Williams ME Pulliam CC Hunter R Johnson TM Owens JE Kincaid J Porter C Koch G The Short-Term Effect of Interdisciplinary Medication Review on Function and Cost in Ambulatory Elderly People J Am Geriatr Soc 2004 52 1 93 98 10.1111/j.1532-5415.2004.52016.x 14687321

[B42] Pacini M Smith RD Wilson EC Holland R Home-based medication review in older people: is it cost effective? PharmacoEconomics 2007 25 2 171 180 10.2165/00019053-200725020-00008 17249858

[B43] Doran E Robertson J Rolfe I Henry D Patient co-payments and use of prescription medicines Aust N Z J Public Health 2004 28 1 62 67 10.1111/j.1467-842X.2004.tb00634.x 15108749

[B44] Central Statistics Office Women and men in Ireland 2011 2012 Dublin, Ireland: Stationary Office Available from: http://www.cso.ie/en/media/csoie/releasespublications/documents/otherreleases/2011/Women%20and%20Men%20in%20Ireland%202011.pdf.

[B45] Barry CA Bradley CP Britten N Stevenson FA Barber N Patients’ unvoiced agendas in general practice consultations: qualitative study BMJ 2000 320 7244 1246 1250 10.1136/bmj.320.7244.1246 10797036 PMC27368

[B46] Department of Health and Children Quality and fairness: a health system for you 2001 Dublin, Ireland: Stationery Office

